# Human Breast Milk: From Food to Active Immune Response With Disease Protection in Infants and Mothers

**DOI:** 10.3389/fimmu.2022.849012

**Published:** 2022-04-05

**Authors:** Gatien A. G. Lokossou, Léonce Kouakanou, Anne Schumacher, Ana C. Zenclussen

**Affiliations:** ^1^Research Unit in Applied Microbiology and Pharmacology of Natural Substances, Polytechnic School of Abomey-Calavi, Department Human Biology Engineering, University of Abomey-Calavi, Abomey-Calavi, Benin; ^2^Department of Immuno-Oncology, Beckman Research Institute, City of Hope National Medical Center, Duarte, CA, United States; ^3^Department of Environmental Immunology, Helmholtz Centre for Environmental Research and Perinatal Immunology, Saxonian Incubator for Clinical Translation, Medical Faculty, University of Leipzig, Leipzig, Germany

**Keywords:** human breast milk, breastfeeding, mother and infant health, immunity, microbiota

## Abstract

Breastfeeding is associated with long-term wellbeing including low risks of infectious diseases and non-communicable diseases such as asthma, cancer, autoimmune diseases and obesity during childhood. In recent years, important advances have been made in understanding the human breast milk (HBM) composition. Breast milk components such as, non-immune and immune cells and bioactive molecules, namely, cytokines/chemokines, lipids, hormones, and enzymes reportedly play many roles in breastfed newborns and in mothers, by diseases protection and shaping the immune system of the newborn. Bioactive components in HBM are also involved in tolerance and appropriate inflammatory response of breastfed infants if necessary. This review summarizes the current literature on the relationship between mother and her infant through breast milk with regard to disease protection. We will shed some light on the mechanisms underlying the roles of breast milk components in the maintenance of health of both child and mother.

## Introduction

Breastfeeding is considered as an optimal way to provide the developmental nutrients needs to newborns and shaping their immune system. Indeed, the immune system of an infant matures by learning to fight infections, establishing adequate tolerance to the environment and the developing microbiota. Breastfeeding is characterized by important psychological consequences on neonates and mothers (1–4).

Breastfeeding is the most optimal food for infants, and in this regard, the World Health Organization (WHO) and the United Nation International Children’s Emergency Fund (UNICEF) recommend exclusive breastfeeding of babies at least during the first six months after birth and add complementary foods in addition to breastfeeding for up to 2 years or beyond (2). In addition to provide nutrients, breast milk is rich in microbiota and non-immune and also immune components to ensure the infant protection against numerous diseases and support maturation of the developing immune system of an infant (5, 6). The roles of the different breast milk components are far from being completely understood. Of note, immune cells such as B and T lymphocytes, regulatory cells, monocytes/macrophages, neutrophils, natural killer cells and IgA, IgG and IgM antibodies are found in the breast milk (7). Breast milk-derived antibodies are produced as a result of pathogenic or non-pathogenic stimulation of mucosa-associated lymphoid tissues in the gut and airways (8). Therefore, breast milk-derived immune components are related to a wide range of intestinal and respiratory pathogens which the infant will encounter (8–10).

Studies in human have shown that many factors such as geography, infant sex or number of pregnancies influence the heterogeneity and composition of human breast milk (HBM) (11). These parameters shape the composition of HBM and therefore impact the maturation and the susceptibility of the human immune system. Indeed, Holmlund et al. have shown that the maternal country of birth influences the pro- and anti-inflammatory contents of HBM, resulting in susceptibility or not to immune-mediated diseases such as allergy or necrotizing enterocolitis (11).

However, breast milk may also transfer virus such as Epstein–Barr virus (EBV) and cytomegalovirus (CMV) from infected mothers to the infants (12), without infection in infants (13–15). Furthermore, as hepatitis B or C virus are lowly presented in breast milk, breastfeeding is not a contraindication by infected mothers (14). Influenza, rotavirus and HIV virus transmitted by breast milk from infected mothers could lead to infant increased disease susceptibility (12, 14, 16). Moreover, environmental chemicals are sometime also found in HBM leading to toxicity (17, 18). All of these environmental factors need to be assessed for a positive impact of HBM on dyad mother/infant.

Furthermore, maternal microbiota, especially breast-derived microbiota (BDM), is one of the relevant features in the development of the immune system of the infant and his microbiota colonization, affecting mucosal and systemic immunity (10). Bacteria strains are shared in the dyad mother/infant (19, 20). BDM is involved in shaping of the microbiota of offspring, resulting in important differences between breast-fed infant microbiota and those of formula-fed infant (21, 22). BDM include mostly *Staphylococcus* and *Streptococcus* bacteria (23), but other bacteria such as *Bifidobacterium*, *Lactobacillus*, and *Acinetobacter* are commonly detected (24, 25). Many factors, namely, geographic locations, maternal lifestyle, the delivery mode and contact to microorganisms influence BDM composition, with variable consequences on the infant immune system (26, 27).

In this review, we summarize the current literature on the breast milk with focus on both non-immune and immune compositions of the breast milk. We also shed some light on their roles in the maintenance of the health of both child and mother. 

## Cells, Microvesicles and Molecules Found in HBM

Breast milk can be subdivided into colostrum, transitional milk and mature milk that reflect the time of breastfeeding from the delivery and the needs for the newborn growth (28). Indeed, colostrum is produced from delivery until seven days while transitional milk is produced between days seven and fifteen after delivery. The mature milk production begins four weeks after postpartum (29). Colostrum is the most important type of HBM based on it high concentration of immune components (7). Overall, the different types of HBM is composed of factors, namely, breast-derived cells, blood-derived cells (30), antibodies, vitamins, and many other cells-derived factors (extracellular vesicles, nucleic acids, enzymes, polysaccharides, lipids, and hormones). All this is reinforced with the presence of probiotic bacterium (10, 31–35). Immune component levels decrease over time and become stable when breast milk is mature (29). Breast-derived cells included lactocytes and myoepithelial cells and also progenitor and stem cells (32). A sustainable proportion of breast milk stem cells is capable of crossing the intestine of nursed infants, entering their circulatory system, and populating distant organs (32, 36, 37). Molès and colleagues have proposed a specific mechanism termed maternal microchimerism (MMC) allowing the transfer and persistent nesting of maternal cells to infant intestinal mucosal tissue, and from there, to other infant immune tissues (38). Milk cells crossing through intestinal mucosal tissue is allowed by weakening interepithelial junctions (39). An important number of breast milk-derived cells are transferred to infant before gut closure and lead to more efficient and effective maturation of mucosal immunity and/or systemic immunity (38).

These natural human milk bioactive components enhanced immunity in the infant during the first years of life (40). High proportion of breast-derived immune cells is activated showing the transfer of active immunity to the infant. These active cells supports the immunological maturation and immune defense of newborns against pathogens (41–43). While it is expected that other components will be identified in the future, the overall picture indicates that HBM may have considerable impact on the dyad mother/infant health.

### Stem Cells

Multicolor flow cytometry has been used to identify in breast milk two major groups of cells, the breast-derived cells, and the blood-derived cells. Both of these groups contain heterogeneous progenitor stem cells, namely, early-stage stem cells, hematopoietic stem cells, mammary stem cells, mesenchymal stem cells, neuro-progenitor cells, and myoepithelial progenitor cells (44–52). Healthy infants consume between 0.5 and 1.5 L of breast milk per day. Immune cells constitute less than 2% of mature human milk-derived cells in healthy mothers. Therefore stem cells ingested daily will represent an important number of breast and blood-derived cells and may reach billions of cells (46, 48, 53). Indeed, in a recent publication, the higher number of hematopoietic stem cells in mature breast milk compared to colostrum was described (53). Moreover, Kakulas et al., formerly Hassiotou, using luminous mice have shown that viable multifunctional stem cells are in breast milk and can cross gastrointestinal (GI) tract mucosa and enter the systemic circulation of the breastfed neonates (48). After crossing the GI tract through diapedesis and entering the bloodstream, these cells immigrate into the different organs and differentiate into functional cells (**Table 1**) (32). Therefore, specific breast-derived stem cells are found in the thymus, liver, pancreas, brain, bone marrow, spleen, intestinal tissues, and lungs and remain unharmed after (32, 48, 54–57). For instance, the incorporation of breast-derived stem cells in the brain is followed by differentiation into the neuron and glial cells (57, 58).

Using gene expression in stem cells (47, 59), Li et al. have recently characterized stem cells in preterm and term mother’s milk (53). They have shown that gestational age at delivery but also body mass index (BMI), the mode of delivery and parity influenced the percentages of hematopoietic stem cells in colostrum and transitional milk. These observations confirmed the Briere et al. data in which breast milk samples obtained from feeding mothers of preterm and term offspring are obviously different in the proportion of stem cells (60). Moreover, stem cell numbers changed dynamically at different breastfeeding stages (53, 59). Mesenchymal stem cells (MSC) (CD44^+^, CD90^+^, and CD105^+^ cells) represent 10–15% of stem cells (50) and MSC were shown to be higher in mature milk than in colostrum (59). These changes in colostrum *vs* mature milk were not confirmed by Shujuan et al. (53), showing the need of more data to better describe the breast milk composition.

It is still a challenge to identify the origin of human breast milk stem cells (hBmSc). Hassiotou and Hartmann have suggested that at least some subpopulations of hBmSCs could have the same origin as hematopoietic stem cells (48). This suggestion is reinforced by the presence of other blood-derived cells in breast milk (51, 61). Unfortunately, the precise mechanisms by which they enter into breast milk and the physiological roles of hematopoietic stem/progenitor cells in early neonate development remain unclear.

The last decades allowed good documentation of the transmission of cells from mother to fetus and *vice versa* through placenta but also from mother to breastfed infants *via* breast milk (36, 37, 62–64). Breast milk-derived cells seem to cause microchimerism in breastfed infants and these cells may survive in the infants for several years as those cells exchanged between mother and fetus *in utero* (37, 65–68). This microchimerism is firmly involved in tissue repair and in the efficient maturation of infant immune system (38, 69), for instance, by inducing tolerance to mother noninherited maternal antigen (NIMA), especially in case of graft from mother to breastfed infants (37). This not fully understood physiological role of hBmSC becomes a great challenge to learn more about all the implications of breastfeeding and harness stem cells to provide significant therapies.

### Innate Immune Cells

At birth, the newborn immunity is characterized by its remarkable difference compared to the adult immune system (70–72) placing an infant at an increased risk of infection (70, 73, 74). However, breastfeeding is perfectly adapted to provide needs to learn how to establish balance between fighting infections and tolerance to harmless environmental compounds (40). Furthermore, in some cases the immune system of the newborn is associated with developmental immune deficiencies which might render the newborn more susceptible to infections, eventually decreases vaccine efficacy and increases the susceptibility of the respiratory and gastrointestinal (GI) tract to infections (75). Breastfeeding will reinforce and educate neonatal immune trajectories allowing the best protection for the newborn (76, 77). In addition to stem cells (53, 54, 59), HBM is rich in immune cells such as monocytes/macrophages, neutrophils, cytotoxic, helper and regulatory T cells, natural killer (NK) cells and B cells. These cells provide active immunity to neonates by their abilities to produce bioactive molecules such as lactoferrin, lysozyme, oligosaccharides, cytokines and others. B cells complete these bioactive molecules by producing immunoglobulins (secretory IgA, IgG and IgM) (29, 30, 78). Breast milk shapes the development of the newborn immune system and is involved in the development of the microbiota of the infant (10). The proportion of leucocytes is significantly higher in colostrum than in transitional milk and in mature milk. More interesting, although the number of leucocytes/ml decreased significantly from colostrum to mature HBM, the variety of leucocytes and their phenotypic characteristics is associated with minor difference with the breastfeeding time, the length of pregnancy or delivery mode (79–82).

#### Macrophages

Macrophages are part of the innate immune system that detect pathogenic microbes or infected cells and trigger immune responses by producing inflammatory mediators and activating adaptive immune responses. These cells are professional antigen presenting cells and therefore important players during adaptive immune activation. Breast milk macrophages (BrMM) represent up to 80% of colostrum and transitional HBM, showing their involvement in the immune protection of the infant (83). Recent data have confirmed the abundance of monocytes/macrophages in HBM and are grouped into inflammatory (CD16^−^) and non-inflammatory (CD16^+^) macrophages (84–87). Although a consistent number of monocytes/macrophages found in breast tissue are originated from the peripheral blood, the gut, or the nasopharynx-associated lymphoid tissues, a fraction is locally constituted in the mammary tissue (8, 78). The strong presence of macrophages in HBM is due to their high phagocytic role (88) and BrMM were able to produce spontaneously granulocyte-macrophage colony-stimulating factor (GM-CSF) upon exposure to HBM components. In the presence of exogenous interleukin-4 (IL-4) alone, BrMM differentiated into CD1^+^ DCs showing their abilities to stimulate T cells (89, 90).

Zheng et al. reported that macrophages homing to breast, and are found in milk, in response to ongoing respiratory infections in the nursing infant, displayed an anti-inflammatory profile that correlated with protection of the infant against mucosal inflammation (84). Indeed, these breast macrophages of a mother are trafficking to the mucosal surfaces of the offspring in mice and other animal models (36, 39, 58, 62, 91). Therefore, it is clear that these cells play a crucial role in the gastrointestinal mucosa and are involved in the maturation of the immune system of the nursing infant without causing inflammation (73, 84, 92, 93). These breast-derived macrophages may therefore exert an important role in innate immunity but also triggering high antigen-specific T cells (89). These findings showed the uniqueness functions of BrMM in both normal and pathological conditions, which is not the case with other differentiated cells that are transferred into breast milk. Moreover, BrMM immunoprotective function is improved by opsonin-independent mechanisms than blood monocytes, by differentiating into lectinophagocytic properties (94). However, the mechanism underlying this function needs to be investigated. 

#### Neutrophils

Neutrophils are important sensor cells that help to prevent infections by blocking, disabling or digesting invading particles and microorganisms. These cells are key players during acute inflammation and also contribute to chronic inflammatory conditions and adaptive immune responses (95).

Neonatal neutrophils are in lower numbers and they are not able to effectively kill bacteria as neonatal neutrophils do not form neutrophil extracellular traps (NETs). Expression of adhesion molecules (e.g., L-Selectin: CD62L, Integrin: MAC-1) is also decreased on neonatal neutrophils, affecting their binding ability to the endothelium. They are also characterized by a lower expression of TLR2 and TLR4 as well as decreased level of phagocytosis, neutrophilic burst, and diminished capacity of degrading intracellular infective agents (96). HBM neutrophils will make up these deficiencies by being more active and motile, and presenting interactive features showing that HBM neutrophils are involved in active immune response in infants (44, 93, 97, 98). During breastfeeding, the relative median frequencies of neutrophils within total leukocytes significantly increased from colostrum to mature milk (79) showing their involvement in infant immune protection. Furthermore, milk from asthmatic mothers has demonstrated elevated proportions and activation of neutrophils. These changes of HBM neutrophils due to maternal asthma may result in alteration in the immune response of the infant (99). Besides, breastfeeding mothers with high levels of neutrophils in their milk (>20%) are more likely to have children who are allergic to cow’s milk than breastfeeding mothers with low neutrophil levels (100).

On the other hand, this does not question the fact that data from the systematic reviews have shown the breastfeeding protection of infants against the development of atopic diseases such as eczema and food and respiratory allergy, especially when there is a family history (101, 102).

#### Natural Killer (NK) Cells

Natural killer cells are important players in the innate immune response. These cells act directly against infection but also produce cytokines that activate others immune cells. Therefore, NK cells contribute to neonate protection (42, 45). Recent data have described NK cells in HBM (30, 44). NK cells represent on average 0.5% of colostrum-derived leucocytes, 1.3% of transitional breast milk cells, and 2% of mature breast milk cells (53). The proportion of NK cells was significantly lower in mature milk of preterm mother (79) and formula-fed newborns (103). Interestingly, Fernandez et al. have shown that human milk-derived *Lactobacillus* may enhance the release of Th1 cytokines, and activate NK cells, and others immune cells (104).

NK cells migrate to the breast from lymphatic vessels and systemic circulation (105), and arrive in mammary glands (106). These cells do not play a protective role only in the infant gastrointestinal tract but also protect the mammary gland from infection (42).

The involvement of breast milk-derived NK and NKT cells in antibody-dependent cellular cytotoxicity (ADCC) and antibody-dependent cellular phagocytosis (ADCP) should be more investigated to better understand how NK cells contribute to pathogen clearance in infant. For instance activated NK cells are found in HBM during CMV reactivation, supporting the immune response against CMV (15).

#### Innate Lymphoid Cells (ILCs)

One of the most recent discoveries in the composition of breast milk is the presence of innate lymphoid cells (ILCs) that often were found near the lining of the aero-digestive tract (96, 107–109). These cells seem to be more activated and/or differentiated than their blood homologous (108) and constituted guard against breast infection by pathogens. They share functions with T cells (110) and may shape neonatal innate immunity (108) as they are important players in intestinal microbiota and the adaptive immunity of the newborn (111–113). Based on cytokines secretion and transcription factor profiles, ILCs were regrouped within 3 subtypes of ILCs with ILC1 counted 3 to 10 times of ILC3 which was 3 times the count of ILC2. More interesting, ILC1 produce higher levels of IFN-γ than IL-5 and IL-22 produced by ILC2 and ILC3 respectively (108). ILC2 and ILC3 are therefore important player in the GI tract health as IL-22 is highly associated with the maintenance of gut barrier integrity (113). Furthermore, ILCs play an important role during inflammation and tissue homeostasis (108, 112). As breast milk-derived ILCs modulate the immunity of the neonate, maternal ILCs may affect the infant ILCs. But it is not clear how this occurs. The next step will be to investigate the role of maternal ILCs in shaping infant ILCs and understand how; 1—they protect the infant from enteric infections and intestine inflammation, 2—they are harnessed to regulate the establishment of the gut microbiome of the infant.

### Adaptive Immune Cells

#### T Lymphocytes

As key actors of adaptive immunity, lymphocytes represent 5 to 10% of all HBM leucocytes (105). Among these lymphocytic populations, T cells represent more than 80% whereas B cells are only 4 to 6% (114). In HBM, CD4^+^ T cells are present in activated state and express activation markers such as CD40L, sCD30, IL-2 receptor, HLA-DR^+^, human mucosa lymphocyte antigen-1 (hMLA-1), or late activation protein-1 (LAP-1). These adaptive immune cells also express CD45RO which is associated with immunological memory functions (115–118). Low number of naïve CD4^+^ T cells were also described in HBM (117). In HBM, 26–76% of CD4^+^ T cells co-express the C chemokine receptor 5 (CCR5) and CXC chemokine receptor 4 (CXCR4), the major co-receptors used by HIV to attach and entry cells (119–121). Although the expression of CD103, the mucosal homing marker indicating their mucosal origin, these HIV target cells were preserved in HIV-infected mothers breast milk unlike in other mucosal immune areas in which these cells were quickly eliminated by HIV (122). Moreover, the HIV-infected women were clinically stable on highly-active antiretroviral therapy (HAART) allowing low or undetectable viral loads but do not achieve CD4^+^ T cell reconstitution in the periphery (122). As HBM contains memory CD45RO^+^ T cells with helper-inducer function, they may be resistant to killing by HIV (123) and shelter the mammary gland from HIV replication (124–127).

HBM-derived CD8^+^ T cells are homed from the mother mucosal immune system to the breast and are presented at a higher proportion than CD4^+^T cells. Indeed, Wirt et al. have shown that the HBM mean CD4/CD8 ratio of T cell was 0.88, ranging from 0.40 to 1.25 whereas it decreases in peripheral blood (116). CD8^+^ T cells express L-selectin, α4β7 integrin, mucosal addressin cell adhesion molecule-1, intestinal homing receptor CD103, mucosal homing receptor CCR9, high level of CD45RO, and HLA-DR markers showing their effector memory functions (117, 128, 129). Comparable to peripheral blood, HBM CD8^+^ T cells express IFN-γ alone or IFN-γ and granzyme together and increase during human cytomegalovirus, HIV, influenza, or EBV infection (80, 130). The increased number of CD8^+^ T cells were positively correlated with breast milk viral load showing that local virus replication induced homing of antigen-specific CD8^+^ T cells into the breast for local viral control and relative lack of viral transmission *via* mammary glands (131). As showed in murine pups, maternal breast milk cytotoxic T lymphocytes home to the Peyer’s Patches of the breastfed infant and this was due to the expression of gut-homing molecules α4β7 and CCR9. Moreover, breast milk cytotoxic T cells have a high capacity to produce potent cytolytic and inflammatory mediators when compared to those generated by the breastfed infant (130). These data suggest the compensation of the learning adaptive immune system of the infant in order to limit oral infectious during the postnatal phase (36).

Since the first description of presence of leucocytes in HBM, especially in colostrum (132, 133), subsequent findings have elucidated the HBM composition. It is established that breast milk is involved in initiating immunologic tolerance in newborns to antigens of both mother and environment to achieve lifelong homeostasis and prevent immune-related disorders (128, 134). It was reported that the thymus size of breast-fed infants is double to that of non-breast-fed infants (135–137) and supports the role of human milk on regulatory T cells expansion during the first three weeks after birth in breastfed human babies. These cells are nearly twice as abundant as in formula-fed babies. These cells also control the immune response of the baby against maternal cells transferred with breast milk and help to reduce inflammation (65, 66, 138, 139).

As the increased number and functionality of T regulatory cells (Treg) are crucial for establishing and maintaining the semi-allograft fetus (140–142), these cells are the main players for providing tolerance in the newborn (138, 139). Cérbulo-Vázquez et al. have demonstrated a higher frequency of CD127^−^ CD25^++^ cells (Treg) in colostrum than peripheral blood, showing a passive transfer of tolerance mediated by memory cells and it is positively associated with vaginal delivery (143).

These tolerogenic cells also exhibited higher expression of CD25, CD152, CD279, and TGF-β than blood-derived Treg cells, suggesting the increased ability of colostrum-derived Treg cells to mediate immunomodulation and maternal microchimerism (37, 143).

Treg cells will play a beneficial role in the newborn by lowing inflammation in the gut mucosa (144) and reducing the risk of developing allergy, asthma, obesity, and diabetes type 1 during the short-and long-term life of the infants (145).

Moreover, it has been shown that maternal allotransplants from the human kidney are better tolerated if the graft recipient has been breastfed during early life (146, 147). This immunosuppressed anti-maternal immunity may be related to the ability of newborn naive CD4^+^ T cells to differentiate toward tolerogenic features and drive the suppression of normal immune responses leading to T cell anergy (148–150). The breakdown of tolerance to maternal antigens mechanism throughout the life can drive autoimmune diseases such as type 1 diabetes (T1D), multiple sclerosis (MS), and systemic lupus erythematous (SLE), suggesting the necessity of maintaining this maternal microchimerism induced by HBM (151–155). More recently, CD161^+^/TRAV1‐2^+^ mucosal‐associated invariant T cells (MAIT) and γδ T cells were found in breast milk. The study by Bedin et al., reported an increased frequency of CD161^+^/TRAV1‐2^+^ MAIT cells in HIV-infected mother breast milk compared to healthy mother (107). Furthermore, γδ T cells have selective compartmentalization and represent almost 9% of CD45^+^ breast milk leucocytes whereas they represent only 2.5% of blood-derived lymphocyte cells. γδT cells number decrease slowly in HIV-infected women breast milk and represent 7.26% of breast milk-derived leucocytes. In blood from HIV-infected cells, 3.31% of CD45^+^ cells were TCR γδ cells (107). This observation shows the detection of unconventional T cells that may also be involved in both the protection against infection of the lactating mammary gland and the maturation of infants’ gut and microbiota (107). One of the future challenges will be to focus on exploring their precise role in gut and microbiota integrity.

#### B Lymphocytes

The most important thing we knew about breast milk is the presence of a variety of immunoglobulins. These antibodies are secreted by B lymphocytes or plasma cells (79).

After memory B cells or Ig-secreting cells were generated following B cells activation, they recirculate through peripheral lymph nodes, spleen, tonsils, and mucosal effector sites such as exocrine glands, including the lactating mammary gland (9, 156–159).

Phenotypically and functionally, two main subsets of memory B cells have been described in HBM (156, 160). The most abundant subset, class-switched B memory cells (CD27^+^IgD^−^) express surface IgG or IgA molecules, while non-class switched B cells, less abundant (CD27^+^IgD^+^) express IgM and are involved in the response to T cell-independent antigens (161, 162).

HBM-derived B cells are activated cells with high levels of CD38 expression, low expression of complement receptors, a feature of plasma cells (163–165). Mammary gland-homed memory B cells and plasma cells arise from other mucosal areas where they have encountered antigens (139–141). These B cell homing-molecule profiles are different from those of homed B cells in nasal-associated lymphoid tissue (NALT) or gut-associated lymphoid tissue (GALT) (166–169). However, HBM-derived memory B cells are α4β7^+/−^, α4β1^+^, CD44^+^,CD62L^−^, sharing their profile with GALT B cells which also bear α4 and β7 integrin chains and are CD62L^−^ (168, 170–172). Based on homing profiles, these observations showed that the breast is more closely associated with intestinal mucosa than with upper respiratory mucosal sites (28). B cell migration to the mammary glands is mediated by chemokine CCL28 which is the mucosa-associated epithelial chemokine (111, 173, 174). Moreover, during lactation CCL28 is upregulated and is linked to the CCR10 receptor expression on IgA-secretory cells (IgA-SCs) in the mammary gland, allowing IgA-SCs accumulation in the mammary gland, unlike to IgG-SCs or IgM-SCs (173, 174). Since IgA-SCs are accumulated in the mammary gland, >90% of Igs secreted in breast milk are IgA (111, 175). The entero-mammary pathway appears to be another process by which the mother could also provide the protection to offspring (176). Indeed, during suckling, the mouth-derived pathogen of the infant may be retro-transferred *via* the nipple to breast inducing a mammary immune response (41, 177, 178). After the recognition of the pathogen by the mother immune system, specific antibodies are then produced in milk and fed to the infected infant (42, 111, 179). Cleary, maternal, and infant infections stimulate a rapid leukocyte response in breast milk (41). Moreover, in the milk of HIV-infected mothers and stools of their breastfed children, the levels of total IgA and IgG were increased indicating the activation and homing into the breast of gut mucosa-derived B cells (175, 180). Notably, breast milk samples and stool samples from HIV-negative and HIV-positive babies breastfed by their HIV-infected mothers, contained high levels of HIV-specific IgG. However, IgA antibodies were less frequent, and IgM are scarce (181). These antibodies, that specifically target gp120, purified from HIV-exposed breastfed infants, were able to inhibit HIV binding to HT29 cells and monocyte-derived macrophages (181). Moreover, the intestinal mucosa of infant exposed to HIV by breastfeeding produces HIV-specific antibodies with inhibitory properties to HIV, showing the effect of prophylactic breastfeeding on immune responses of an infant against natural immunization with HIV (181).

It is therefore clear that class-switched B memory cells may highly be involved to compensate the low antigen-presenting capacity of newborn macrophages.

Acquired immunity of a newborn is characterized by its excellent capacities to learn fighting pathogenic microbes and during this learning phase, it is supplemented by maternal antibodies (70–72, 182). These antibodies are provided first *in utero* and after birth by breast milk. These *in utero* maternal IgGs are actively transported through the placenta to the fetus *via* the Brambell receptor commonly called FcγRn (183). However, IgG with certain specificities and with high avidity are more efficiently transferred to the fetus than others (184). These IgG antibodies are involved in newborn protection and mediate antimicrobial and antibody-related cytotoxicity (185). These antibodies recognize microbes on intestinal mucosa but also in the circulation and tissues (43, 186).

Soluble milk IgAs also have antimicrobial properties and are higher in milk from very preterm delivered mothers compare to term milk (187–190) and in preterm milk *vs* term milk (185). sIgA could inhibit pathogen binding (79, 191) by complexing antigens and taking them up by intestinal DCs which will then process and present these antigens (192). IgM, antimicrobial antibodies, are also found in breast milk and mediate cytotoxicity (185).

### Extracellular Microvesiles

Recently, extracellular vesicles (EV) have been identified in breast milk and seem to shape the breastfed infant immune system, especially his intestinal immune response (10, 124, 193, 194). Moreover, Hock et al. have shown that rat milk-derived exosomes promote intestinal epithelial cell viability, enhance proliferation, and stimulate intestinal stem cell activity. These findings support the fact that milk-derived exosomes play important roles in gut health and prevent necrotizing enterocolitis in infants (195).

Breast milk-derived EVs contain a rich cargo, namely, cytosolic and membrane proteins, mRNA, miRNA, and are oriented towards cell-to-cell communication and result in the immune shaping of newborns (196–198). EVs include two subsets such as microvesicles (100–1.000 nm) (199, 200) and exosomes (30–100 nm) (201, 202). Exosomes differ from the other cellular microvesicles according to **1—**the endocytic pathway by which they are generated, **2—**their homogenous cup-shaped structure, when observed by electron microscopy, and **3—**their buoyant density ranges between 1.13 and 1.19 g/ml on a sucrose gradient (203). Although the protein composition of exosomes varies from their originating cell, proteome analyses shown that the tetraspanins, CD9, CD63, CD81, and the endosomal sorting complexes required for transport (ESCRT)-related proteins, Alix and TSG101, are constituents of nearly all exosomes and are markers used for the detection of exosomes (196, 203). The acetylcholine esterase (AChE) activity is also a useful tool to control for exosomes isolation and purification (204). Exosomes, are formed through the endosomal pathway and are released from cells following the fusion of multivesicular bodies with the plasma membrane (205). Exosomes are released by a large range of cells, namely, immune cells, neural cells, stem cells, placenta cells, and many cancer cells (reviewed here (205)), and can be isolated from different body fluids, such as serum, urine, cerebrospinal fluid, amniotic fluid and breast milk (10, 195, 203, 205–207). There are enriched by immunologically relevant components that direct immune responses (208). High concentration of exosomes is found in colostrum compared with mature milk. HLA-DR is highly incorporated in early milk-derived exosomes which displayed significantly lower levels of HLA-A, -B, and -C in relation to mature milk. Indeed, breast milk-derived exosomes phenotype depends on maternal sensitization and lifestyle, which might increase allergy sensibility in the offspring (209). Comparatively to breast milk volume, a high level of micro RNAs (miRNAs) is found in breast milk (196). These large various non-coding molecules (210) are stable in an acidic conditions of GI tract (211) and regulate post-transcriptional expression of genes and have biological activities in humans (210, 212, 213). According to the HBM immune cells miRNAs and mammary gland cells miRNAs, HBM-derived miRNAs seem to originate from immune-related and mammary gland cells (193). HBM-derive exosomes contain mostly miR-148a-3p, miR-22-3p, miR-200a-3p, miR-146b-5p, miR-30d-5p, let-7a-5p, miR-30a-5p, let-7f-5p, let-7b-5p, and miR-21-5p (193, 214–221). Upon in the GI tract, these microvesicles-derived microRNAs are taken up by intestinal cells (222). Moreover, some of these miRNAs seem to shape the neonate immune system (210, 214) by limiting cytokine production (223) and inhibiting T-cell proliferation induced by DCs (223) or B cell activation, TLR4 signaling, and macrophage activation (193, 224).

Since gestational age induces changes in the miRNA profile which influence metabolism and lipid biosynthesis (225), it becomes opportune to understand the impact of these changes on breast milk composition and offspring health (193). In the same way, other HBM exosome-derived miRNAs induced B- and T-cell activation, proliferation, and differentiation (193). They are able to protect newborn from infection by reducing inflammation and boosting the immune response by many ways (193, 222, 226).

Carr et al. have recently proposed that breast milk miRNAs may act as TLR7/TLR8 agonists and may boost the immune system (193). This opens the way for a better understanding of the specific role of HBM exosomes-derived miRNAs on the immune system and the neonatal microbiota (10, 193, 227). 

## Cytokines/Chemokines Profile of Breast Milk

HBM contains an array of cytokines and chemokines reviewed by Nolan et al. (228). These cytokines are primarily originated from the mammary glands, and include IL-1 β (229, 230), IL-2 (229), IL-4 (230), IL-5 (230), IL-6 (84, 229–231), IL-8 (232), IL-10 (189, 230), IL-12 (233), IL-13 (230, 233), TNFα (229, 231), TGF (transforming growth factor)-β (232), IFN-γ (235), granulocyte-colony stimulating factor (G-CSF) (234), monocytes chemotactic protein 1 (MCP-1) (115) and Regulated upon Activation, Normal T Cell Expressed and Presumably Secreted (RANTES) (93, 230). Purified immune cells from HBM have been shown to be able of producing several cytokines (231). For instance, ongoing infection in breastfeeding infant change milk-derived macrophage profiles into a more anti-inflammatory profile compared to the healthy breastfeeding infant (84). It will be very worthwhile to generate more data to understand how these cytokines stay stable and passage through the infant digestive system. These molecules may be embedded and protected until they reach the intestine (234, 235). The HBM-derived cytokines shape the maturation and development of immune cells in infants. For instance, maternal milk-derived TGF-β, IL-6, and IL-10 induce the development and differentiation of IgA-producing cells (230) and maturation of the naive intestinal immune system (236). Moreover, infant infection is associated to IL-6 and IL-8 increase in HBM that also contains CCL2 and CX3CL1 (84).

Many factors influence the cytokine/chemokine profile of breast milk. For example, geographic location, migration of women to a place different from the place of birth, the number of pregnancies, etc., determine the cytokine/chemokine profile of HBM. Thus, studies have shown that maternal country of birth may influence breast milk composition of cytokines. For instance, immigrant mothers from low income country with high exposure to pathogens, to low exposure developed country, continue to produce higher levels of pro-inflammatory cytokines (IL-6 and IL-8) and TGF-β in their breast milk when they gave birth in developed country (237). In countries with high pathogens exposure, it is relevant to produce higher levels of TGF-β1 and IL-6 in breast milk because of their involvement in stimulation of IgA synthesis (238, 239). Therefore, their offspring will be primed for more inflammatory cells to fight pathogens. However, when these mothers immigrate to developed countries with low exposure to pathogen, their infants continue to be primed with higher levels of IL-8, IL-6 and TGF-β1, resulting to higher incidence of inflammatory disease (237).

The number of pregnancies also influences the levels of cytokines in breast milk. Indeed, a larger number of previous pregnancies are associated to lower levels of IL-6, IL-8 TGF-β and soluble CD14 in breast milk (237), resulting in a global damping of the immune system. A lot of information is still needed in order to better appreciate the role of these cytokines/chemokines.

## HBM Microbiota

Immune development during infancy is in part related to bioactive factors provided by HBM. Among these factors, probiotic bacteria are important players and their variation due to geographic locations, maternal lifestyle, the delivery mode and contact to microorganisms can impact positively or negatively on the infant immune system (26, 27). Breast milk microbiota contains a large number of specific bacterial species with antimicrobial properties and health benefits (240–242). These bacteria include beneficial, commensal and probiotic bacteria (243, 244).

In regard to the number of bacteria consumed per day, about 8.10^5^ bacteria/day, breast milk appear to be the second source of bacteria for the infant after normal delivery (21). Human breast milk-derived bacteria spectrum in healthy mothers is highly diverse. The major bacterial species belong to *Lactobacillus* (*L. gasseri*, *L. fermentum*, *L. plantarum*, *L. rhamnosus*, and *L. salivarius*) (25, 241, 245, 246) and *Bifidobacterium* (*B. breve*, *B. bifidum*, *B. lungum*, *B. dentium*, and *B. adolescentis*) (247, 248). Among *Staphylococcus* spp. presented in HBM*, S. epidermidis* was predominant while *S. aureus* was also found (249, 250). *Streptococcus* spp. included mostly *S. salivarius, S. hominis*, and *S. mitis* (249, 250). Recent analysis of HBM using sequencing technologies has provided more detail about the diversity of microbiota of milk with more than hundred bacterial species (10). Interestingly, these bacteria are not present at the same lactation time and their proportion among all bacterial is not identical in all women. It is therefore believed that variations in HBM microbiota are based on maternal diet, genetic, the mode of delivery, demographic or environmental differences (27, 251–253). Indeed, during the first month of lactation, full-term and preterm milk samples shared bacteria such as *Bifidobacterium*, *Lactobacillus*, *Staphylococcus*, *Streptococcus*, and *Enterococcus* with significantly lower number of *Bifidobacterium* spp. in preterm group (254). However, Urbaniak et al. did not found significant changes in the bacterial composition between term and preterm patients (255), showing that more research are needed to highlight the composition of breast milk with regard to the gestational age. According to the mode of delivery, cesarean sections were associated with higher overall bacterial concentration in colostrum and transitional milk. Moreover, higher concentration of *Streptococcus* spp. and lower concentration of *Bifidobacterium* spp. were found in cesarean sections than vaginal deliveries (254).

Anti-biotherapy during pregnancy could also influence microbiota diversity, leading to a significant decrease of *Bifidobacterium* and *Lactobacterium* spp (256).. Other factors that might impact breast milk microbiota are mother BMI and geographical locations. Indeed, high BMI is associated with lower bacterial diversity with reduction of *Bifidobacterium* spp. levels and this lower bacterial diversity is correlated to celiac disease in mothers (257). Mothers living in Africa, Asia, and Europe have different milk profiles (253, 258), even if *Staphylococcus* and *Streptococcus* were constant compounds of the milk microbiota regardless the geographical locations (253, 258).

Too much debate surrounded the origin of breast milk bacterial population. Similarities were found between the adult skin microbiome and milk microbiota, suggesting bacterial transfer from the skin of the mother during infant suckling (21, 242, 259). In addition, infant oral cavity also influences the breast milk bacterial composition based on retrograde back flow mechanism (179, 260, 261). This mechanism referred to a “retrograde transfer” of external bacteria, and the gut-mammary pathway for translocation of internal bacteria (261). Many data are expected from BDM analysis and the harnessing of BDM will be useful in disease prevention and therapy.

## HBM and Infectious Diseases

Mortality and risk of infection in breastfed infants *vs.* nonbreastfed infants is one of the markers of immunological benefits of breastfeeding (262). Indeed, Victora et al. meta-analysis shows that breastfeeding during the first six months of life is strongly protective against infectious diseases (101). Their analysis and others have demonstrated at least 88% reduction of mortality in breastfed compared to nonbreastfed infants (101, 263). Regarding the many advantageous anti-infection and immunological properties described in this review, HBM appear as the optimal food with strong potential to reduce infectious diseases and to optimize the well-being of offsprings, especially in premature neonates. Although, mishandling or misadministration of HBM may result in transmission of infection to neonate, the many immune compounds prevails the potential risk of infection. A recent study showed that mothers are able to significantly increase their breast milk-derived leucocytes in response to acute neonatal infection (41). Some chronic diseases and respiratory infections and also otitis and gastroenteritis rates are shown to be decreased in breastfed infants (264, 265). HBM is also associated with decreased risk of necrotizing enterocolitis and late-onset sepsis in premature neonates (266, 267). When mothers are infected by airbones diseases such as measles, varicella and tuberculosis, infants are recommended to be temporarily separated from their mother. Because these airbone diseases are not transmitted by their milk as long as there are no lesions in the breast area, infants can receive pressed milk from their mothers (268). Chronically infected mothers by hepatitis B virus can produce in their breast milk some virus even if the risk of transmissiom is low (269). Therefore, the benefits of breastfeeding prevails the risk of transmission and it is recommended for mothers to continue breastfeeding (270, 271). In case of Herpes simplex virus (HSV) which is responsible of perinatal infections and, even if less frequently in neonate, HSV infections result from infected maternal breast lesions. As there is no evidence of HSV transmission in HBM, breastfeeding or pumping milk in the absence of breast lesions is recommended when women are infected by HSV (268).

Some pieces of evidence suggest that HBM can provide immune protection against Severe Acute Respiratory Syndrome novel Coronavirus 2 (272, 273). Indeed, significant specific IgA to the full SARS-CoV-2 spike protein and significant IgA, IgG, and/or IgM directed to SARS-CoV-2 spike protein receptor-binding domain are found in HBM (272, 273). As SARS-CoV-2 RNA is expressed in HBM (274) without it being possible to demonstrate the viability and transmissibility of detected virus (275–277), the feeding of HBM should be encouraged, either *via* direct breastfeeding or *via* pumped HBM.

Acute gastroenteritis may be cause by norovirus (NV) resulting in diarrhea in infant. It has been shown that high positivity rates and titers of IgA directed to NV in HBM is associated with reduced diarrheal symptoms in NV infected infants (278). These data confirm the antiviral properties of HBM and taking more attention to breask milk composition will help fighting infectious disease in low income countries.

HBM is an antibacterial and anti-infectious food which reduces the possibility of pathogens transmission to neonate (270). These anti-infection properties are confirmed in *in vitro* studies that showed that HBM-derived *Lactobacillus rhamnosus* and *Lactobacillus crispatus* have anti-infection activities against *Staphylococcus aureus* (*S. aureus*). As *S. aureus* is involved in mastitis in lacting women (279), antibiotic-resistant nosocomial infections, and neonatal infections, breastfeeding is and important weapon to reduce the pathogen load owing to *S. aureus* (270, 280). Moreover, *in vitro* and *in vivo* studies in mice have shown that HBM-derived *lactobacilli* strains, especially, *Lactobacillus salivarius*, have antibacterial activity and that inhibit *Salmonella enterica* adhesion to mucins. This results in high survival proportion in infected mice (240, 280). In addition, lactic acid bacteria in HBM allow acid pH, resulting in inhibition of the growth of anaerobic bacteria and protection of the gut (240, 281).

*Escherichia coli* (*E. coli*) are responsible of neonatal systemic infection and urinary tract infection. However, HBM is not a source of *E. coli* due to the anti-infection properties of breask milk (282). Therefore, if a mother is experiencing *E. coli* infection, breastfeeding migh not be interrupted (270). HBM is also known to prevent oropharyngeal infant *Haemophilus influenza* type b (Hib) colonization. Therefore, breastfeeding migh be continued with careful masking and hand hygiene when mother is experiencing Hib disease (270, 283).

During delivery, the risk of transmission of Group B *Streptococcus* (GBS) is significant, resulting in neonatal late-onset sepsis. Although GBS can be found in HBM (284), the anti-infection properties of HBM justify the fact that breastfeeding is encouraged during GBS disease. However, in case of recurrent late-onset GBS disease in preterm neonate, it suggests avoiding breastfeeding until the disease is resolved (285).

Sexually transmitted diseases caused by *Chlamydia* or *Neisseria gonorrhea* can result in perinatal infection. Chlamydial acquired infection during delivery results in neonatal conjunctivitis and pneumonitis. While HBM is not a source of chlamydial transmission, specific secretory IgA has been found in colostrum and mature milk of infected women allowing the continuation of breastfeeding (286). *N. gonorrhea* also is not transmitted by HBM and breastfeeding is recommended if women experiencing infection are treated with appropriate medication (286).

While tuberculosis (TB) remains a burden in developing countries, there has been no demonstrated case of TB transmission through HBM, excepted during TB mastitis (287). Therefore, during separation between TB experienced woman and infant, infants are allowed to receive pumped milk with adequate precautions and are breastfed in case of asymptomatic TB (279). 

## Global Benefit for Breastfeeding Mothers

The benefit of breastfeeding in both mother and infant health is demonstrated by recent data showing that HBM bacteriome are associated with anti-tumor properties (280). Indeed, live, heat-killed and cytoplasmic fractions of HBM-derived *Enterococcus faecalis* and *Staphylococcus hominis* have shown anti-tumor activity against a breast cancer cell line *in vitro* (288).

*In vitro* study has also demonstrated that *Lactococccus lactis* has therapeutic effects against colon cancer (289). HBM bacteriome has also relevant implication in mastitis that occurs in about 30% of breastfeeding women (290, 291). HBM-derived *Lactobacillus salivarius* and *Lactobacillus gasseri* are shown to be effective substitutes for the treatment and control of mastitis (292).

A collaborative study analyzing 47 epidemiological studies, including women with breast cancer and without the disease, has demonstrated an inverse relationship between breastfeeding and breast cancer. A reduction of 4.9% in the incidence of breast cancer is associated with the duration of breastfeeding (293). This observation was confirmed in a meta-analysis performed by Victora et al. who found that longer breastfeeding is associated with 7% reduction of breast cancer (101). Ovarian cancer is also reduced by 30% when infant was breastfed for a long duration (294).

Potential mechanisms underlining the relationships between breastfeeding and cancers may include inflammatory disorders and epigenetic reprogramming in the tissues, resulting in disorders in progenitor cell pools. For instance, in breast cancer, high *FOXA1* methylation is found among women who did not breastfeed (295). Using technological advances, it will be possible to shed light on the mechanism(s) by which breastfeeding contributes to reduce specific cancers.

Compelling data have demonstrated the maternal health outcomes of breastfeeding. Victora et al. have well demonstrated how breastfeeding acts as natural contraceptive by spacing birth, especially when mother exclusive breastfed (101, 296).

Type 2 diabetes (T2D) risk is also reduced by breastfeeding (297, 298), while every 6 months an increase in breastfeeding duration is associated to 1% lower mean body maternal index (299). The mechanism underlining the benefit effect of breastfeeding includes the increase in the number of β cells into the pancreas. Indeed, during breastfeeding prolactin induces serotonin in β cells improving insulin secretory function and glucose tolerance (300). The impact of prolactin on the immune system has been described during the last 2 decades. This polypeptide can act as hormone when synthetized by hypophysis and as cytokine when it is provides by neurons, mammary epithelium, skin, prostate, immune organs, and cells (301). In addition to controlling lactation and maternal behaviors, polymorphisms in prolactin gene appear to play an important role in autoimmune disease including SLES (301, 302). Indeed, hyperprolactinemia impairs B-cell tolerance (303), and results in SLE (303). 

## HBM and Infant’s Immune Diseases

T1D characterized by the destruction of pancreatic B cells (304), is strongly based on genetic susceptibility but also involves non-genetic factors (305–307) including breastfeeding which could protect against T1D in infants (308). T1D risk is reduced in newborn breastfed during more than 3 months or during minimum of 2 weeks exclusive breastfeeding after postpartum (309) while T1D risk is twofold increased in nonbreastfed infants (307). However, this study did not found positive association between the time of introduction of specific solid foods and T1D (307). In contract two important studies, the Diabetes and Autoimmunity Study in the Young (DAISY) (310) and the Type 1 Diabetes Prediction and Prevention Project (DIPP) (311) suggested that introduction of solid foods before 4 months and after 6 months of age increase the risk of T1D (303). The risk of T2D in childhood is also reduced by proper breastfeeding including the duration of breastfeeding and exclusive breastfeeding (312).

The protective role of breastfeeding in immune disease, including juvenile idiopathic arthritis (JIA) and rheumatoid arthritis (RA) is controversial (303). For instance, breastfeeding has been associated to protection against JIA (313) whereas other authors did not support this observation (314). It also has been shown that the gut microbiome is altered among JIA patients suggesting a role of breastfeeding (315). Futures studies are needed to better understand the impact of HBM on the risk of JIA.

The influence of HBM on the risk of developing RA is not clear (303) and a recent meta-analysis has concluded that the risk of RA is not affected by the duration of breastfeeding (316). 

## Controversy on HBM Protection or Susceptibility to Allergy and Other Immune Diseases

Most studies on the relationship between breast milk and allergy development in offspring often show contradictory results (317–320). It has been proposed that breastfeeding is associated with anti-allergic properties due to it bacteriome. For instance, HBM probiotic *lactobacilli*, especially *Lactobacillus gasseri* together with *Lactobacillus coryniformis*, decrease the frequency and severity of allergic responses to cow milk proteins in animal studies (321). Moreover, some studies have demonstrated the reduction of infant eczema between one and two years when mother are supplemented with *Lactobacillus* spp. and/or *Bifidobacterium* spp (322, 323). However, the supplementation with probiotic in the first six months after birth did not show a reduction of the risk to develop atopic eczema (324).

According to Victoria et al., there is no clear consistent evidence indicating a protective effect of breastfeeding towards either eczema or food allergy (101). More data are therefore needed to clearly understand the role of breastfeeding in allergy prevention.

Other immune disease such as Celiac disease (CD), inflammatory bowel diseases (IBD) and MS seem to be reduced by breastfeeding (303). Indeed, breastfed infants who have developed CD have better long term health (325) owing to the later introduction of gluten (326). Likewise, IBD (327) including ulcerative colitis and Crohn disease (328) is found to be reduced by breastfeeding (327). However, in other studies, no association was found between breastfeeding and IBD (329, 330). This controversy is based on difference in the definition of exclusive or nonexclusive and duration of breastfeeding (303). Breastfeeding during at least 4 months has been associated to protection against MS (152) whereas the impact of breastfeeding duration on the risk of developing MS continues to be questioned by others (331, 332).

## Conclusion and Future Perspectives

The current understanding of HBM supports that it is biologically and temporally adapted to the breastfeed child. Breast milk composition varies greatly from one pregnancy to another, from one mother to another, and despite these differences, breast milk remains the best food for the infant. Immune benefits associated to breastfeeding are well documented, but the role of some immune cells is still unclear and need to be investigated. Moreover, how these immune cells especially effector memory cells and some innate immune cells such as macrophages are involved in infant protection against infection and immune-related diseases need to be more investigated. As stem cells are also present in breast milk and are able to differentiate into various organs, it become ambitious to consider breast milk-derived stem cell use in transplantation.

Overall, understanding mechanisms under HBM components in newborn protection and wellbeing may increase the promotion and support of breastfeeding to achieve the third sustainable Development Goal of The United nations, namely, maternal and child health. This third sustainable goal also includes non-communicable disease such as breast cancer, overweight, obesity, and diabetes. It is noteworthy that breastfeeding is not only relevant for nutrition, a part of the second Development Goal, but also for education which is a part of the fourth Goal. Therefore, breastfeeding can effectively contribute to the reduction of poverty. 

## Author Contributions

LAG conceived and wrote the manuscript. LK, AS, and ACZ edited the manuscript and figures. All authors listed have made a substantial, direct, and intellectual contribution to the work and approved it for publication. 

## Conflict of Interest

The authors declare that the research was conducted in the absence of any commercial or financial relationships that could be construed as a potential conflict of interest.

## Publisher’s Note

All claims expressed in this article are solely those of the authors and do not necessarily represent those of their affiliated organizations, or those of the publisher, the editors and the reviewers. Any product that may be evaluated in this article, or claim that may be made by its manufacturer, is not guaranteed or endorsed by the publisher.

## Figures and Tables

**Table 1:** Infant’s organs in which specific cells of HBM are found.

Infant distal organs	Breast milk cells			
	Progenitor cells	Stem cells	Hematopoietic stem cells	Immune cells
Brain	+	+	–	–
Bone marrow	+	+	+	–
Intestinal tissues	+	+	–	+
Liver	+	+	–	+
Lungs/spleen	+	+	+	+
Pancreas	+	+	–	+
Thymus	+	+	+	–

+ indicates the presence of HBM progenitor cells, stem cells, immune cells or hematopoietic stem cells.

## Abbreviations

HBM, human breast milk; BDM, Breast-derived microbiota; EBV, Epstein–Barr virus; CMV, cytomegalovirus; HIV, human immunodeficiency virus; MMC, maternal microchimerism; BMI, body mass index; GI, gastrointestinal; MSC, mesenchymal stem cells; hBmScs, human breast milk stem cells; NIMA, noninherited maternal antigen; EV, extracellular vesicles; ESCRT, endosomal sorting complex required for transport; AchE, acetylcholine esterase; HLA, human leucocyte antigen; DCs, dendritic cells; BrMM, breast milk macrophage; GM-CSF, granulocyte-macrophage colony-stimulating factor; IL, interleukin; CD, cluster of differentiation; NET, neutrophile extracellular traps; MAC-1, macrophage-1 antigen; ADCC, antibody dependent cellular cytotoxicity; ADCP, antibody dependent cellular phagocytosis, ILCs, innate lymphoid cells; HAART, highly-active antiretroviral therapy; Treg, T regulatory cells; TGF, tumor growth factor; MAIT, mucosal-associated invariant T cells; NALT, nasal-associated lymphoid tissue; GALT, gut-associated lymphoid tissue; SCs, secretory cells; IFN, interferon; TNF, tumor necrosis factor; G-CSF, granulocyte-colony stimulating factor, MCP-1, monocytes chemotactic protein 1, RANTES, Regulated upon Activation, Normal T Cell Expressed and Presumably Secreted; SLE, Systemic lupus erythematosus; T1D, Type 1 Diabetes; T2D, Type 2 Diabetes; DAISY, Diabetes and Autoimmunity Study in the Young; DIPP, Prediction and Prevention Project; JIA, Juvenile idiopathic arthritis; RA, Rheumatoid arthritis; CD, Celiac disease; IBD, Inflammatory bowel diseases; MS, Multiple sclerosis.
